# Can Psychological Expectation Models Be Adapted for Placebo Research?

**DOI:** 10.3389/fpsyg.2016.01876

**Published:** 2016-11-28

**Authors:** Winfried Rief, Keith J. Petrie

**Affiliations:** ^1^Department of Psychology, Philipps University MarburgMarburg, Germany; ^2^Department of Psychological Medicine, University of AucklandAuckland, New Zealand

**Keywords:** expectation, placebo, nocebo, prediction error, expectation violation, associative learning

## Abstract

Placebo responses contribute substantially to the effect and clinical outcome of medical treatments. Patients' expectations have been identified as one of the major mechanisms contributing to placebo effects. However, to date a general theoretical framework to better understand how patient expectations interact with features of medical treatment has not been developed. In this paper we outline an expectation model that can be used as framework for experimental studies on both placebo and nocebo mechanisms. This model is based on psychological concepts of expectation development, expectation maintenance, and expectation change within the typical paradigms used in placebo research. This theoretical framework reflects the dynamic aspects of the interaction between expectations and medical treatment, and offers a platform to combine psychological and neurophysiological research activities. Moreover, this model can be used to identify important future research questions. For example, we argue that the dynamic processes of expectation maintenance vs. expectation changes are not sufficiently addressed in current research on placebo mechanisms. Therefore, the question about how to change and optimize patients' expectations prior to treatment should be a special focus of future clinical research.

## Introduction

Placebo mechanisms contribute substantially to clinical outcome in many fields of medicine (Schedlowski et al., 2015). In randomized clinical trials, patients receiving placebo treatment typically achieve results that are almost equivalent to the response of the active intervention group. This has been shown not only for patient reported outcomes, such as pain and depression, but also for objectively assessed biological parameters such as immune reactions (Schedlowski et al., 2015), cardiovascular reactions (Meissner, 2008), or polysomnographic assessments of pain and sleep variables (Winkler and Rief, 2015).

Expectations have been identified as one of the major components contributing to placebo reactions (Schwarz et al., 2016). If patients have a need for medical interventions, they are exposed to stimuli in the clinical setting that trigger specific treatment- and outcome expectations. These stimuli include the nature of the treatment itself—such as surgery, medicines, or injections. They also include the characteristics of the clinician and the relationship formed with the patient as well as the doctor's confidence in the therapy and explanation of the treatment. The wider treatment context such as the reputation of the facility and status of the clinic may also impact on treatment outcome expectations. As these are all factors that operate psychologically to enhance or decrease the placebo response, expectation theories can contribute to a better understanding of placebo effects. In this paper, we will use the terms expectation and expectancy interchangeable, although expectancy is more frequently used when also including implicit expectations and implicit expectation effects.

Atkinson's expectancy-value theory outlines that behavior in challenging situations is predicted by the interaction of prior expectations to be able to manage such a challenge successfully and the subjective value of the specific task (Atkinson and Reitman, 1956). In the health setting, the value of the challenge is typically associated with the hope to survive the illness and to reduce the burden caused by its pain and symptoms. According to the theory, a better clinical outcome is predicted if the expected improvements caused by a treatment are of high personal value and patients have a strong self-belief to be able to cope with the situation (self-efficacy). Indeed, low expectations of specific self-efficacy, and low expectations of therapy-driven improvements result in low treatment adherence (Horne and Weinman, 2002).

A further relevant background theory is “prospect theory” developed by Kahneman and Tversky (1979). This theory emphasizes the subjectivity of the definition by which an outcome can be considered as gains vs. losses. The authors highlight the fact that potential losses are frequently more relevant for behavioral decisions than expected gains. Applying this theory to the clinical context, patients' anxieties, and concerns about treatment can be more relevant to predict their behavior than the expected benefits of their treatment.

Expectations are frequently developed through a process of associative learning. An important model predicting how repeated trials of associative learning can lead to learned reactions is Rescorla-Wagner's model, which has been principally developed to explain Pavlovian conditioning effects (Rescorla, 1967). This model has also substantial relevance for understanding the development and the consequences of expectations. The power of an expectation corresponds in part to the associative strength in the formula of this model. Accordingly, the strength of expectation is dependent on the number of trials confirming these associations and/or the learning rate. Additionally, the model also postulates that expectations can eventually achieve a maximum level that limits further increases in association. Learning is reconceptualized as a change of expectations. Therefore, the discrepancy between expected outcome and experienced outcome is a major precondition to initiate learning processes. The important contribution of this model is for the understanding how expectations are modified. The Rescorla-Wagner Model became one of the basic concepts that stimulated the development of paradigms investigating prediction and prediction error effects in neuroscience (Schwarz et al., 2016).

While this selective collection of psychological theories on expectation is not comprehensive, it illustrates that these psychological theories have been developed with a strong non-clinical focus. Therefore, we want to develop a theoretical framework for expectation effects in the clinical context, that offers a platform to integrate these psychological theories with empirical approaches that will help explain placebo and nocebo effects in the context of medical treatments.

## Adapting the ViolEX-model

Recently, we developed a general model that conceptualizes how expectations influence various outcomes in clinical psychology, and when expectation violations lead to a change vs. a persistence of expectations (“the ViolEx-model;” Rief et al., 2015). The original model was developed as a broad theoretical framework to better understand the dynamic interactions between expectation effects, expectation violations, and their feedback loops to modify expectations in general. Here, we adapt this model to placebo and nocebo research and clinical encounters.

**Figure 1:**
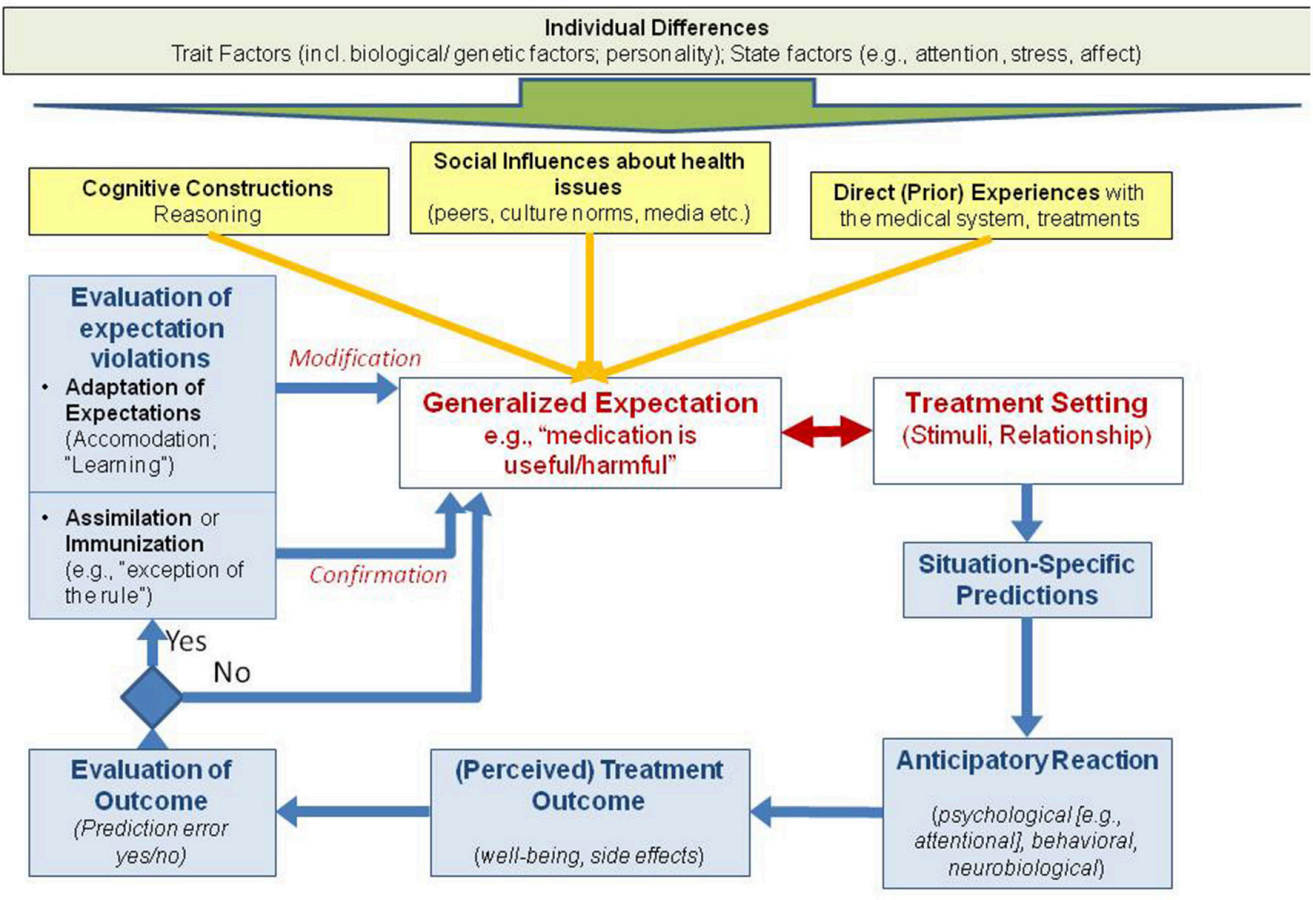
**Adaptation of the VIOL-EX model for placebo research**.

The core of the model in Figure 1 is the interaction of expectations and clinical situations, such as visiting a doctor for the treatment of bothersome symptoms. This interaction results in predictions, outcome, and outcome evaluations that either confirm or disconfirm pre-existing expectations. The model is complemented by adding trait factors, past learning processes, and state factors to better understand how expectations developed. Different aspects of the model are covered below.

Placebo effects occur when a medical treatment and its context trigger specific expectations about a positive therapeutic outcome. Pre-existing optimistic expectations can amplify the positive effects of treatments (placebo effects), but negative expectations can also induce adverse treatment effects, such as side effects or the absence of treatment-typical improvements (nocebo effects).

The interaction of pre-existing generalized expectations and medical setting variables leads to situations-specific predictions that are associated with typical anticipatory reactions. When a treatment outcome is perceived, an individual evaluates whether it corresponds to the predicted outcome, or whether the outcome is unpredicted, such as when side effects occur. The more frequently expected positive outcomes occur then the generalized expectations are more stable, although this learning process is asymptomatic according to the Rescorla-Wagner-Model (“confirmation,” see Figure 1). If the expected outcome does not occur, or additional unexpected outcomes develop, this will typically lead to a modification of expectations due to expectation-violating experiences (“modification,” see Figure 1). However, it would not be adaptive if individuals were to change their expectations just because of one disconfirming event.

In reality, many people stick to their expectations despite contradictory experiences (e.g., persistence of stereotypes about population groups despite positive experiences with members of them). In the clinical context, the change of expectations is a crucial aspect, although this aspect has been poorly investigated. Patients do not show up in clinical settings without any treatment expectations, but these are not fully concordant with what doctors would like them to expect about their treatment. Therefore, it is quite typical that there is a conflict between patients' expectations (and fears) of a treatment vs. doctors' beliefs about the same therapy. Effects of self-generated expectations are usually stronger than expectations induced from outside the individual (Acosta, 1982; Kemper et al., 2012; Gaschler et al., 2014). The clinical task is thus not to establish new expectations in “naïve” patients, but to change and optimize pre-existing treatment expectations in patients.

Three factors contribute in particular to the development of expectations (see Figure 1, yellow connections). These are prior experience with the health care system (associative learning), social influences about health issues that are established via prior observations or learned indirectly from significant others or through media sources such as the internet. The third process that contributes is the individual personal construction of assumptions as well as the direct instructions received from others. As an example, observational learning is also of central relevance in the clinical context. Patients often have contact or observe other patients, be it in the waiting room of an outpatient clinic, or in a typical inpatient setting. These other patients can either praise or model the improvements from treatment, discuss the skill of a particular doctor, or they can complain about unwanted effects of interventions. The observation of such behavior has been shown to influence the results of the observing patient's treatment (Colloca and Benedetti, 2009; Voegtle et al., 2013; Faasse et al., 2015a).

Most of the associations indicated in Figure 1 are also influenced by pre-existing trait factors (e.g., genetic factors, personality factors), but also by state factors such as selective attention or current options for memory retrieval. Expectancy discrepant effects can lead to a “surprise-attention link” with a shift of attention, which can facilitate or hinder learning processes (Horstmann, 2015).

The “individual differences” mentioned on top of Figure 1 should be interpreted as a dimension influencing most other processes on all levels of this model. The effect of expectations can be also different depending whether they are self-generated vs. cue-induced expectations (Gaschler et al., 2014), with physician's interventions representing more cue-induced expectations. In part, this can help to explain why some physician-induced expectations are less powerful than patient-generated expectations.

## Examples of empirical results to components of the expectation model

The most simple way to induce specific patients' expectations is by offering instructions about expected outcomes. In placebo research, this is typically done by informing patients that a placebo intervention is supposed to be a pain killer (Pollo et al., 2001; Bingel et al., 2011). This effect can be further amplified by inducing positive prior experiences with this specific treatment. Manipulated feedback can also induce expectations that (placebo) treatments can induce strong intervention effects.

Associative learning paradigms using Pavlovian conditioning have been used to demonstrate influences on expectations, not only in pain (Colloca and Benedetti, 2006), but also in various other conditions. Using a similar design, we were able to show that patients can “learn” to develop side effects if they received several applications with the antidepressant amitriptyline, even if eventually the drug is switched to a placebo pill (Rheker et al., 2016). Further, many people have learned that effective drugs are associated with some side effects that indicate the drug is working or powerful. This led to work showing that so called “active placebos” simulating drug-typical side effects induce more powerful placebo responses than “passive placebos” (Moncrieff et al., 2004; Rief and Glombiewski, 2012; Benedetti et al., 2013).

Generalized expectations about medical treatments are not only able to predict positive outcome, but also to predict the development of side effects and other negative outcomes (Faasse and Petrie, 2013). Promoting negative expectations can even abolish the pain-relieving effects of powerful opioids, such as remifentanil (Bingel et al., 2011). Negative beliefs about medicine predict the development of more side effects (Nestoriuc et al., 2010). This can take the form of a general belief that an individual is highly sensitive to the effects of medication in general or sensitive to specific type of medication (Horne et al., 2013; Faasse et al., 2015b). For example, expectations about developing medication side effects for endocrine therapy following breast cancer predicts more problems after treatment onset (Nestoriuc et al., 2016).

The context and environment that the medical treatment is administered is of relevance, in particular, if it includes stimuli that can activate expectations because of prior stimuli-associated experiences. The treatment context can further amplify the effect of positive expectations, e.g., if it is considered to be very professional, friendly, and clean. Treatment-context conditions are also able to influence the reactions to antidepressant drugs, and can even trigger negative effects of antidepressants compared to placebo (Rief et al., 2016). A special aspect of the treatment context is the relationship between therapist and patient. While a positive therapeutic relationship can predict successful treatment outcome, a negative therapeutic relationships can also facilitate the development of adverse treatment effects (Kaptchuk et al., 2008; Koudriavtseva et al., 2012). Moreover, the quality of the therapeutic relationship further predicts patients' adherence, and this association can also contribute to a positive outcome.

In experimental pain research, it has been shown that situation-specific predictions of pain or pain relief activate brain areas that facilitate the expected perceptions (Koyama et al., 2005). When selecting actions (such as drug intake), the brain pre-activates the representation of the predicted consequences (Waszak et al., 2012).

Further biological and psychological pathways of action of specific intervention predictions have been described (Schedlowski et al., 2015). Of particular relevance is also the role of selective attention. If patients expect adverse experiences, they also focus their attention to the specific side effect expected, which increases the perception intensity and facilitates the reporting of adverse outcomes in general (Barsky et al., 2002) or specific to the type of expectations generated (Crichton et al., 2014). Attentional processes themselves can be the result of learning (Mackintosh, 1975; Kruschke, 2003).

If the outcome is as positive as expected, this leads to a confirmation of expectations consistent with the Rescorla-Wagner Model; there is no change in association strength, hence no learning. However, if expectations are not confirmed, it remains unclear how the person will deal with that fact. Several treatment approaches actually set out to induce expectation violations (e.g., exposure therapy in anxiety disorders; Craske et al., 2014; Craske, 2015). However, not every expectation violation subsequently leads to expectation changes. Frequently, patients activate cognitive-attributional assimilation or immunization strategies to weaken or eliminate the expectation violation. The result of successful exposure sessions and other intended expectation violations can be devaluated with cognitions such as: “this was the exception to the rule;” “this only works if a therapist is close to me.” A side-effect free day can still confirm side effect expectations via attributions like: “if I didn't get side effects today, I will probably get them tomorrow.” While these assimilation and immunization processes have been extensively studied in social psychology, an examination of their role in clinical research is still in a very early stage.

The dynamic process of expectation development, maintenance, and change in the clinical context is further influenced by biological and psychological trait and state factors. Genetic aspects can predict whether a person is prone to develop side effects (Wendt et al., 2014), as well as whether a person is prone to develop placebo responses (Hall et al., 2012). Anxiety as a personality factor is able to predict the development of somatic symptoms as a reaction to medical interventions, but also has potential as a predictor of symptom development caused by expected environmental influences (Petrie et al., 2005; Page et al., 2006; Witthöft and Rubin, 2013; Crichton et al., 2014). The current level of biological stress reactions can further influence the interaction of the components of our model. These are just a few examples that the model presented in Figure 1, although already elaborated, is still an approximation, and simplification of the various influences that determine the interaction between expectations and treatment settings.

## Conclusion

The effect of the interaction between patients' expectations and treatment context depends on past experiences, and they are characterized by dynamic interactions that happen during and after the treatment encounter. Most components are also influenced by biological and psychological individual differences such as genetic, personality, and state factors. In total, this model offers a theoretical framework that helps to communicate and connect the different approaches on placebo and nocebo research, both on a more basic scientific level and in terms of clinical applications. It also helps to identify research areas needing more work. One of the specific conclusions we want to draw is that more research is needed how to modify pre-existing expectations in situations where patients' pre-treatment expectations are non-adaptive. Therefore, the focus of research has to move from mere inductions of specific expectations to a better understanding of processes of expectation persistence, expectation violation, and expectation change.

## Author contributions

All authors listed, have made substantial, direct, and intellectual contribution to the work, and approved it for publication. Both authors contributed equally to this manuscript.

### Conflict of interest statement

The authors declare that the research was conducted in the absence of any commercial or financial relationships that could be construed as a potential conflict of interest.
